# Effect of Probiotic Supplementation on Cognitive Function and Metabolic Status in Mild Cognitive Impairment and Alzheimer's Disease: A Meta-Analysis

**DOI:** 10.3389/fnut.2021.757673

**Published:** 2021-12-08

**Authors:** Xurui Li, Chang Lv, Jinxiao Song, Jianguo Li

**Affiliations:** ^1^Department of General Medicine, Hebei General Hospital, Shijiazhuang, China; ^2^Department of Emergency, Hebei General Hospital, Shijiazhuang, China

**Keywords:** Alzheimer's disease, biomarker, meta-analysis, mild cognitive impairment, probiotic, efficacy

## Abstract

**Background:** Alzheimer's disease (AD) is a progressive and multifactorial neurodegenerative disease accounting for 80% of dementia worldwide.

**Objective:** To assess the influence of probiotics on cognitive function in patients with mild cognitive impairment (MCI) and AD.

**Methods:** PubMed, Embase, and Cochrane Library databases were searched for relevant studies.

**Results:** Six randomized controlled trials involving 462 patients with MCI and AD were included in this meta-analysis. The probiotic administration had favorable effects on homeostasis model assessment–insulin resistance [HOMA-IR; Weighted mean difference (WMD) = −0.34, 95% confidence intervals (95% CI): −0.44 to 0.24, *P* < 0.001, *I*^2^ = 0%], very low–density lipoprotein levels (VLDL; WMD = −3.71, 95% CI: −6.11 to −1.32, *P*=0.002, *I*^2^ = 57.7%), quantitative insulin sensitivity check index (QUICKI; WMD = 0.01, 95% CI: 0.00–0.01, *P* = 0.003, *I*^2^ = 51%), and triglyceride levels (WMD = −15.65, 95% CI: −27.48 to −3.83, *P* = 0.009, *I*^2^ = 63.4%) in patients with AD. However, after Hartung-Knapp adjustment, all effects were non-significant except for HOMA-IR (MD = −0.34, 95%CI = −0.58 to −0.11). The changes in the Mini-Mental State Examination, repeatable battery for the assessment of neuropsychological status, and other biomarkers of oxidative stress, inflammation, and lipid profiles (high-sensitivity C-reactive protein, malondialdehyde, and total cholesterol) were negligible.

**Conclusion:** The findings suggested that the consumption of probiotics had favorable effects on the HOMA-IR in patients with AD. However, the probiotic treatment did not affect cognitive function, other biomarkers of oxidative stress, and other lipid profiles.

## Introduction

Alzheimer's disease (AD) is a progressive and multifactorial neurodegenerative disease that accounts for 80% of dementia globally, especially in people over 60 years of age. Clinically, AD is characterized by severe impairments in memory, cognition, and motor function, resulting in decreased mental, behavioral, and functional activities that affect the quality of daily life (1). Contrary to AD where other cognitive skills and the ability to live independently are affected, mild cognitive impairment (MCI) is characterized by deficits in memory that do not significantly impact daily functioning (2). According to the Epidemiological Survey of the Global Burden of Disease Study 2016, ~43.8 million people worldwide had AD in 2016 (3). According to the prediction of the world Alzheimer's report 2015, the total number of patients with AD will reach 74.7 million by 2030 and 131.5 million by 2050, posing a global health challenge (4).

Intestinal microbiota is a group of microorganisms found in the gastrointestinal tract, which plays a key role in anatomy, physiology, and immune host function (5, 6). During the aging process, the composition and function of intestinal microbiota change significantly, which may affect health and cause age-related diseases (7, 8). Recently, the concept of gut–brain axis has emerged, which refers to the two-way relationship between gut and brain, linking gut microbiota with age-related neurodegenerative diseases such as AD (9–11).ssss The interaction between gut and brain involves a complex network of endocrine, immune, and neurotransmitters, which is considered a key target for treating brain health and neurodegenerative diseases (12–14).

In recent years, several studies have evaluated the effect of probiotics in MCI and AD (15–20). Although some researchers have reported that probiotic supplementation can improve cognitive function, the relevant data are still rare, and the results of various studies are inconsistent. Therefore, the purpose of the present meta-analysis was to provide quantitative results of the effect of probiotics on the cognitive function in patients with MCI and AD.

## Materials and Methods

### Literature Search

This meta-analysis was prepared and conducted in accordance with the Preferred Reporting Items for Systematic Reviews and Meta-Analyses (PRISMA) Guidelines (21). PubMed, Embase, and Cochrane Library databases were used to browse and search for reports on the effect of probiotics in patients with MCI or AD. The search focused on studies involving human participants and published in English before February 14, 2021. The references of the identified research and review papers were manually explored to avoid missing any relevant studies. The following keywords and medical terms were used alone or in combination: (probiotics OR probiotic) AND (mild cognitive impairment OR MCI) AND (Alzheimer's disease OR AD). A detailed search string for PubMed is presented in Supplementary Table 1.

#### Selection Criteria

Two independent authors reviewed all relevant studies. Any disagreements were resolved by consensus with a third investigator. The inclusion criteria were as follows: (bib1) randomized controlled trials (RCTs) including patients with MCI and AD; (2) patients in the trial group treated by probiotics and patients in the control group using no probiotic interventions; (3) the mean and standard deviation provided for at least one outcome; and (4) the study with the largest sample size selected for inclusion if more than one study was published with the same patients. The exclusion criteria were as follows: (1) letters, reviews, abstracts and case reports; (2) non-randomized trials; (3) experimental studies of animal models or cell lines; (4) studies published in languages other than English; and (5) lack of accurate data.

#### Data Extraction and Quality Assessment

The following data were extracted from the selected studies: study author, year of publication, study design, country in which the study was conducted, sex, mean age, intervention, duration of probiotic therapy, and outcome of the evaluation. The authors used the Cochrane Collaboration tool (22) to independently assess the risk of bias as “low,” “high,” or “unclear” in terms of performance bias, selection bias, detection bias, reporting bias, consumption bias, and other biases. We used the Grading of Recommendations Assessment, Development and Evaluation (GRADE) approach (23) to assess the certainty of the evidence. Data extraction and quality assessment were performed by two independent authors. Any disagreements were resolved by consensus with a third investigator.

#### Statistical Analyses

All statistical analyses were performed using Stata 16 software (Stata Corporation, TX, USA). Weighted mean difference (WMD) was used to measure data. Heterogeneity was calculated using Higgins (*I*^2^) test statistics. *I*^2^ values 0–25% indicated no heterogeneity, 26–50% indicated low heterogeneity, 51–75% indicated medium heterogeneity, and more than 75% indicated high heterogeneity (24). In case of significant heterogeneity (*P* < 0.10 or *I*^2^ > 50%), a random-effects model was used. Otherwise, the fixed-effects model was used. Since the number of included studies was small and the heterogeneity was high, Hartung-Knapp adjustment was used to produce more robust estimates of variance (25). In addition, funnel plots and Begg's and Egger's tests were used to assess the publication bias (26). A *P* < 0.05 indicated a significant difference.

## Results

### Literature Search and Study Characteristics

**Figure 1:**
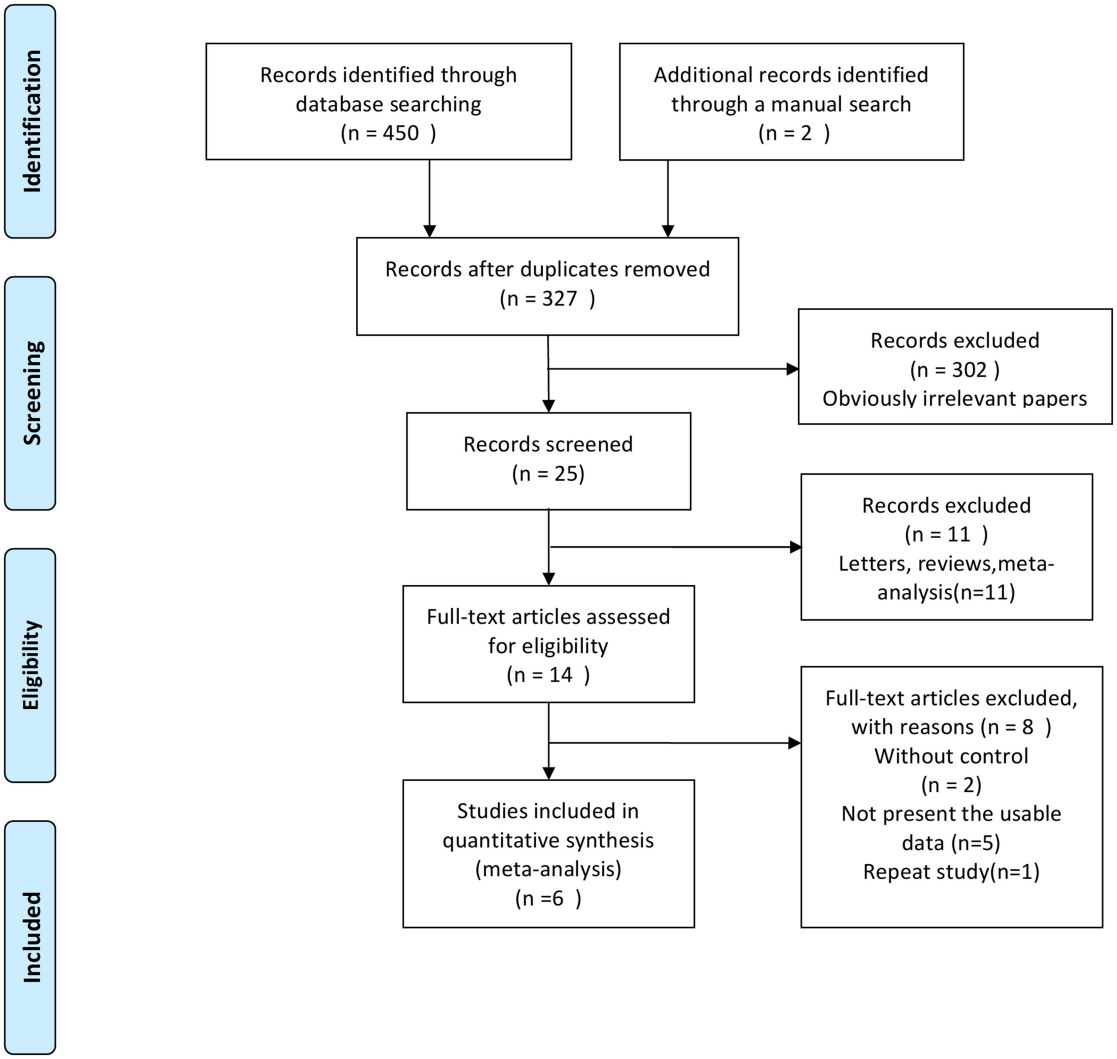
Flow diagram of the study selection process.

**Figure 2:**
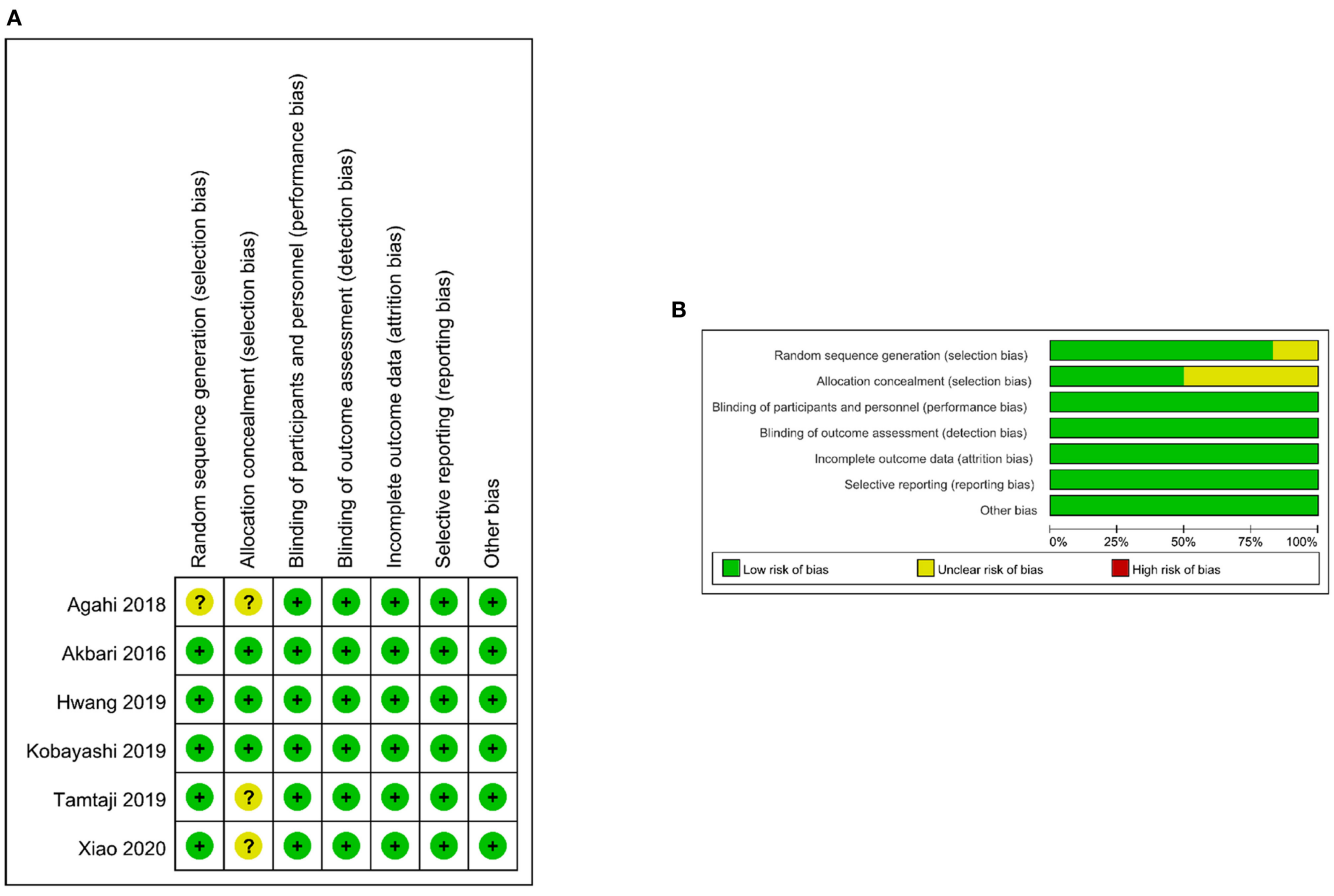
Risk-of-bias assessment for the randomized trials included in the meta-analysis. **(A)** Risk-of-bias summary. **(B)** Risk-of-bias graph. Symbols: (+), low risk of bias; (?), unclear risk of bias; (−), high risk of bias.

A flowchart describing the study selection process is shown in Figure 1. Six studies (15–20) were included in the present meta-analysis. The eligible studies were published between 2016 and 2020, with a total sample of 462. The average age of the participants ranged from 60.9 to 82 years. The main features of the included studies are shown in Table 1. All six studies were randomized, with most (*n* = 5) describing randomization methods. Half of the studies (*n* = 3) did not describe the method of allocation concealment in enough detail. In general, all studies were of high quality (Figure 2). The GRADE assessment of the certainty of the evidence showed that almost all endpoints estimates were derived from moderate quality evidence, and only one had low quality evidence (Table 2).

**Table 1:** Characteristics of the studies included in this meta-analysis.

**References**	**Country**	**Sex (Male%)**	**Mean age**	**Intervention**		**Duration of probiotic therapy**	**Study design**	**Outcomes assessed**
				**Exp**	**Con**			
Akbari et al. (16)	Iran	20%	Exp: 77.67 ± 2.62 Con: 82 ± 1.69	Milk containing a mixture of probiotics (*N* = 30)	Milk (*N* = 30)	12 w	RCT	High-sensitivity C-reactive protein, homeostasis model assessment–insulin resistance, MMSE, plasma malondialdehyde, plasma triglycerides, quantitative insulin sensitivity check index, total cholesterol, and very low–density lipoprotein
Agahi et al. (15)	Iran	35.4%	Exp: 79.7 ± 1.72 Con: 80.57 ± 1.79	A mixture of probiotic bacteria (*N* = 25)	Placebo (*N* = 23)	12 w	RCT	Plasma malondialdehyde
Hwang et al. (17)	Korea	34%	Exp: 68 ± 5.12 Con: 69.2 ± 7	DW2009 (*N* = 50)	Placebo (*N* = 50)	12 w	RCT	Total cholesterol
Kobayashi et al. (18)	Japan	49.6%	Exp: 61.5 ± 6.83 Con: 61.6 ± 6.37	*Bifidobacterium breve A1* (*N* = 61)	Placebo (*N* = 60)	12 w	RCT	High-sensitivity C-reactive protein, MMSE, plasma triglycerides, RBANS, and total cholesterol
Tamtaji et al. (19)	Iran	NA	Exp: 76.2 ± 8.1 Con: 78.8 ± 10.2	Probiotic plus selenium (*N* = 27)	Selenium (*N* = 26)	12 w	RCT	High-sensitivity C-reactive protein, homeostasis model assessment–insulin resistance, MMSE, plasma malondialdehyde, plasma triglycerides, quantitative insulin sensitivity check index, total cholesterol, and very low–density lipoprotein
Xiao et al. (20)	Japan	48.7%	Exp: 61.3 ± 7.7 Con: 60.9 ± 6.9	Probiotic (*N* = 40)	Placebo (*N* = 40)	16 w	RCT	RBANS

*Con, Controls; Exp, Experimental; MMSE, Mini-Mental State Examination; NA, not available; RBANS, repeatable battery for the assessment of neuropsychological status; RCT, randomized controlled trial; w, weeks*.

**Table 2:** Summary of findings table.

**Effect of probiotic supplementation on cognitive function and metabolic status in MCI and AD**						
**Patient or population:** patients with MCI and AD **Settings:** outpatient **Intervention:** probiotic supplementation **Comparison:** no probiotic interventions						
**Outcomes**	Illustrative comparative risks* (95% CI)		**Relative effect (95% CI)**	**No of Participants (studies)**	**Quality of the evidence (GRADE)**	**Comments**
	**Assumed risk**	**Corresponding risk**				
	**No probiotic interventions**	**Probiotic supplementation**				
**Mini-Mental State Examination** Follow-up: 0–12 weeks	The mean mini-mental state examination in the control groups was **0.6**	The mean mini-mental state examination in the intervention groups was **1.08 higher** (0.34 lower to 2.5 higher)		230 (3 studies)	⊕⊕⊕⊖ **moderate**^a^	
**Repeatable battery for the assessment of neuropsychological status** Follow-up: 0–15 weeks	The mean repeatable battery for the assessment of neuropsychological status in the control groups was **5.3**	The mean repeatable battery for the assessment of neuropsychological status in the intervention groups was **6.01 higher** (4.85 lower to 16.86 higher)		196 (2 studies)	⊕⊕⊕⊖ **moderate**^a^	
**High-sensitivity C-reactive protein** Follow-up: 0–12 weeks	The mean high-sensitivity c-reactive protein in the control groups was **0.4**	The mean high-sensitivity c-reactive protein in the intervention groups was **1.31 lower** (3.31 lower to 0.69 higher)		234 (3 studies)	⊕⊕⊕⊖ **moderate**^a^	
**Homeostasis model assessment–insulin resistance** Follow-up: 0–12 weeks	The mean homeostasis model assessment–insulin resistance in the control groups was **0.2**	The mean homeostasis model assessment–insulin resistance in the intervention groups was **0.34 lower** (0.44 lower to 0.24 higher)		113 (2 studies)	⊕⊕⊕⊖ **moderate**^a^	
**Malondialdehyde** Follow-up: 0–12 weeks	The mean malondialdehyde in the control groups was **−0.07**	The mean malondialdehyde in the intervention groups was **0.5 lower** (1.1 lower to 0.1 higher)		161 (3 studies)	⊕⊕⊖⊖ **low**^a, b^	
**Triglycerides** Follow-up: 0–12 weeks	The mean triglycerides in the control groups was **−2.5**	The mean triglycerides in the intervention groups was **15.65 lower** (27.48 to 3.83 lower)		234 (3 studies)	⊕⊕⊕⊖ **moderate**^a^	
**Total cholesterol** Follow-up: 0–12 weeks	The mean total cholesterol in the control groups was **−4.8**	The mean total cholesterol in the intervention groups was **0.05 higher** (0.29 lower to 0.39 higher)		326 (4 studies)	⊕⊕⊕⊖ **moderate**^a^	
**Very low–density lipoprotein** Follow-up: mean 0–12 weeks	The mean very low–density lipoprotein in the control groups was **−0.6**	The mean very low–density lipoprotein in the intervention groups was **3.71 lower** (6.11 to 1.32 lower)		113 (2 studies)	⊕⊕⊕⊖ **moderate**^a^	
**Quantitative insulin sensitivity check index** Follow-up: 0–12 weeks	The mean quantitative insulin sensitivity check index in the control groups was **−0.0075**	The mean quantitative insulin sensitivity check index in the intervention groups was **0.01 higher** (0 to 0.01 higher)		113 (2 studies)	⊕⊕⊕⊖ **moderate**^a^	

* *The basis for the **assumed risk** (e.g., the median control group risk across studies) is provided in footnotes. The **corresponding risk** (and its 95% confidence interval) is based on the assumed risk in the comparison group and the **relative effect** of the intervention (and its 95% CI)*.

***CI:** Confidence interval*.

*GRADE Working Group grades of evidence **High quality:** Further research is very unlikely to change our confidence in the estimate of effect. **Moderate quality:** Further research is likely to have an important impact on our confidence in the estimate of effect and may change the estimate. **Low quality:** Further research is very likely to have an important impact on our confidence in the estimate of effect and is likely to change the estimate. **Very low quality:** We are very uncertain about the estimate*.

a *Unclear allocation concealment*.

b *Unclear random sequence generation method*.

### Quantitative Synthesis

#### Mini-Mental State Examination

**Figure 3:**
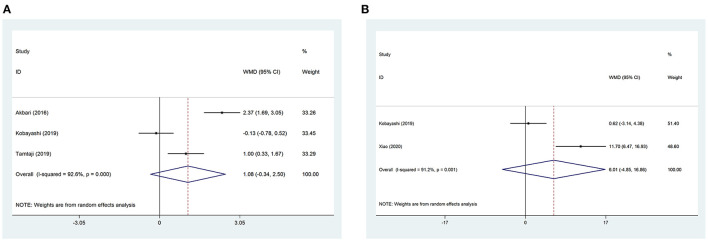
Forest plots showing the effect of probiotic administration on cognitive function. **(A)** MMSE; **(B)** RBANS.

Data regarding Mini-Mental State Examination (MMSE) were reported in three studies. No significant differences were found between the trial and the control group (WMD = 1.08, 95% CI: −0.34 to 2.50, *P* = 0.137, *I*^2^ = 92.6%) (Figure 3A). After Hartung-Knapp adjustment due to few studies included in the analysis, the results were still non-significant (MD = 1.08, 95%CI = −2.03 to 4.19).

#### Repeatable Battery for the Assessment of Neuropsychological Status

Data regarding repeatable battery for the assessment of neuropsychological status (RBANS) were reported in two studies. No significant differences were found between the trial and the control group (WMD = 6.01, 95% CI: −4.85 to 16.86, *P* = 0.278, *I*^2^ = 91.2%) (Figure 3B). After Hartung-Knapp adjustment, non-significant differences were still shown (MD = 6.01, 95% CI = −64.36 to 76.37).

#### High-Sensitivity C-Reactive Protein

**Figure 4:**
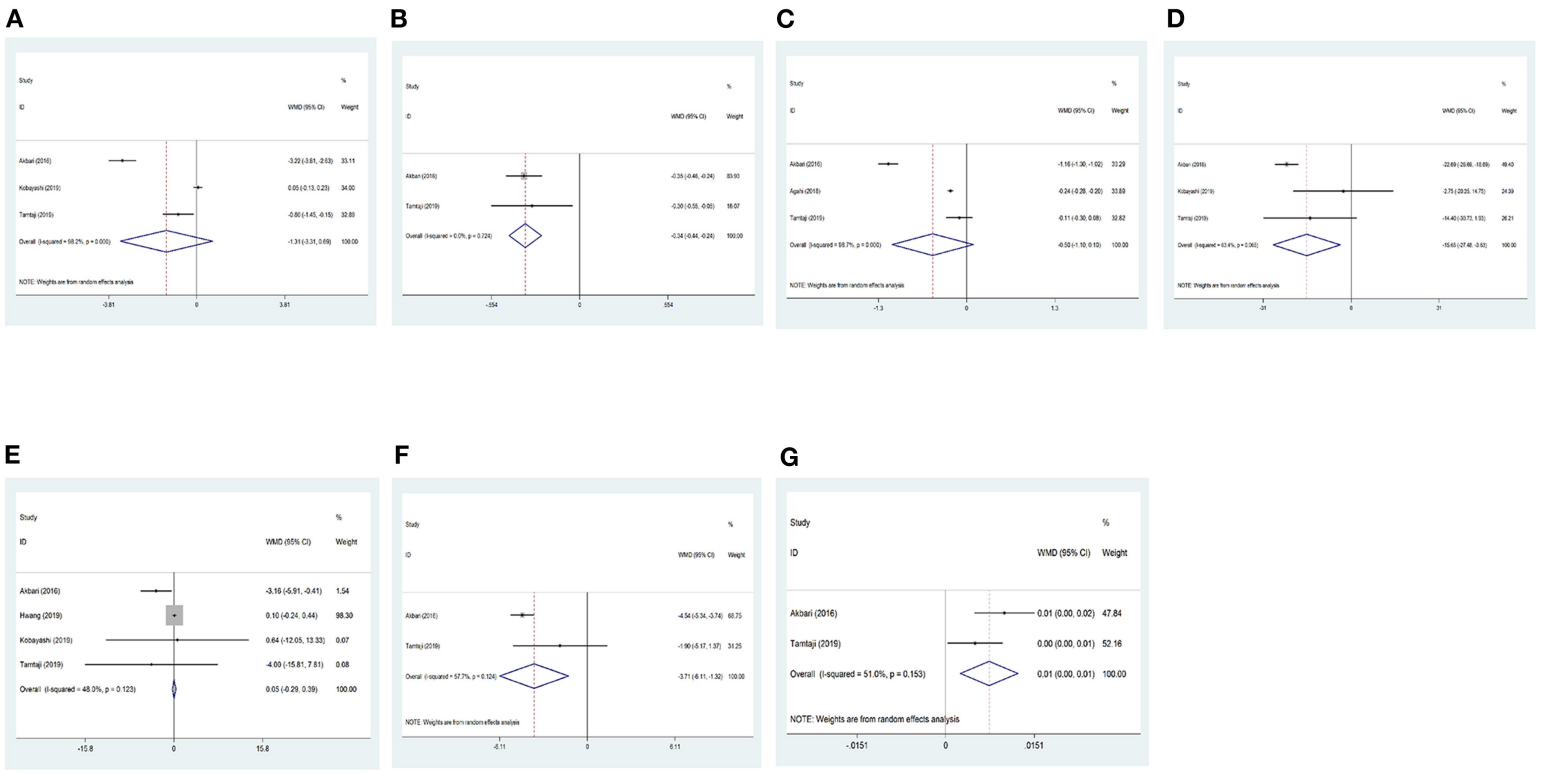
Forest plots showing the effect of probiotic administration on the level of clinical biomarkers. **(A)** hs-CRP; **(B)** HOMA-IR; **(C)** MDA; **(D)** triglycerides; **(E)** total cholesterol; **(F)** VLDL; and **(G)** QUICKI.

Data regarding high-sensitivity C-reactive protein (hs-CRP) were reported in three studies. No significant differences were found between the trial and the control group before Hartung-Knapp adjustment (WMD = −1.31, 95% CI: −3.31 to 0.69, *P* = 0.198, *I*^2^ = 98.2%) (Figure 4A), and after Hartung-Knapp adjustment (MD = −1.31, 95%CI = −5.53 to 2.91).

#### Homeostasis Model Assessment–Insulin Resistance

Data regarding HOMA-IR were reported in two studies. The results showed that homeostasis model assessment–insulin resistance (HOMA-IR) were significantly reduced in the trial group compared with the control group (WMD = −0.34, 95% CI: −0.44 to 0.24, *P* < 0.001, *I*^2^ = 0%) (Figure 4B). However, after Hartung-Knapp adjustments, no significant differences were seen between the two groups (MD = −0.34, 95%CI = −0.58 to −0.11).

#### Malondialdehyde

Data regarding malondialdehyde (MDA) were reported in three studies. No significant differences were found between the trial and the control group before Hartung-Knapp adjustment (WMD = −0.50, 95% CI: −1.10 to 0.10, *P* = 0.101, *I*^2^ = 98.7%) (Figure 4C), and after Hartung-Knapp adjustment (MD = −0.5, 95%CI = −1.92 to 0.92).

#### Triglycerides

Data regarding triglycerides were reported in two studies. The results showed that triglyceride levels were significantly reduced in the trial group compared with the control group (WMD = −15.65, 95% CI: −27.48 to −3.83, *P* = 0.009, *I*^2^ = 63.4%) (Figure 4D). However, after Hartung-Knapp adjustment, the results became non-significant (MD = −15.73, 95%CI = −40.31 to 8.84).

#### Total Cholesterol

Data regarding total cholesterol were reported in three studies. No significant differences were found between the trial and the control group before Hartung-Knapp adjustment (WMD = 0.05, 95% CI: −0.29 to 0.39, *P* = 0.788, *I*^2^ = 48%) (Figure 4E), and after Hartung-Knapp adjustment (MD = −1.22, 95%CI = −4.3 to 1.86).

#### Very Low–Density Lipoprotein (VLDL)

Data regarding VLDL were reported in two studies. The results showed that VLDL levels were significantly reduced in the trial group compared with the control group (WMD = −3.71, 95% CI: −6.11 to −1.32, *P* = 0.002, *I*^2^ = 57.7%) (Figure 4F). However, non-significant differences were observed between the two groups after Hartung-Knapp adjustment (MD = −3.71, 95%CI = −19.26 to 11.83).

#### Quantitative Insulin Sensitivity Check Index

Data regarding quantitative insulin sensitivity check index (QUICKI) were reported in two studies. QUICKI was significantly increased in the trial group compared with the control group (WMD = 0.01, 95% CI: 0.00–0.01, *P* = 0.003, *I*^2^ = 51%) (Figure 4G), but no significant differences were seen after Hartung-Knapp adjustment (MD = −0.01, 95%CI = −0.02 to 0.04).

### Publication BiasN

**Figure 5:**
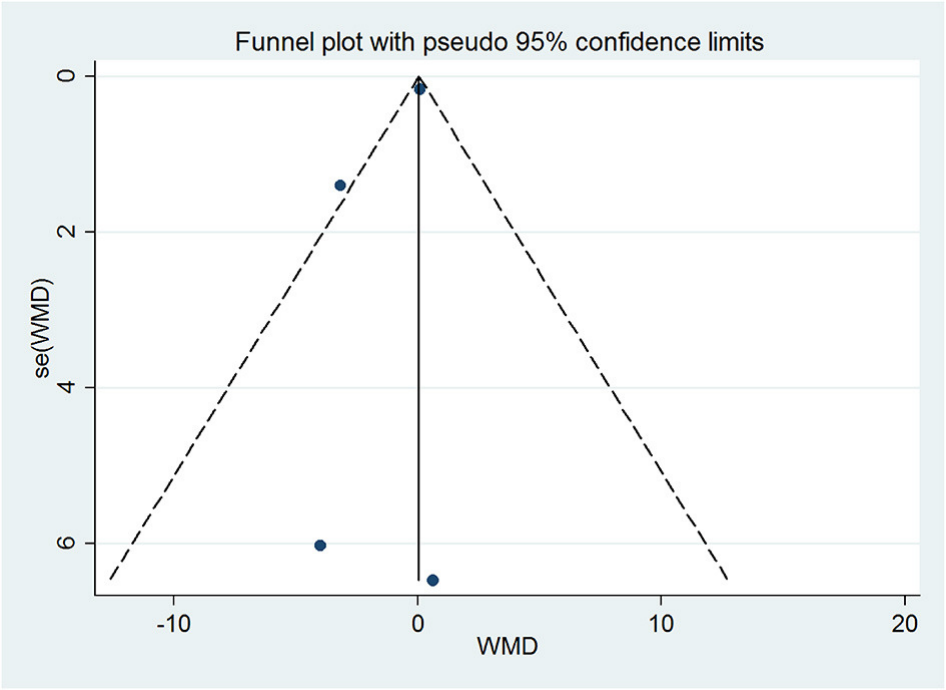
Funnel plot for publication bias test of total cholesterol. Each point represents a separate study for the indicated association.

Publication bias across the included studies was assessed using the funnel plot (Figure 5) and Begg's and Egger's tests. The results for total cholesterol (Begg's test *P* = 1.000, Egger's test *P* = 0.342) showed no evidence of publication bias.

## Discussion

All included studies were RCTs, which further improved the preciseness and credibility of this meta-analysis. The present study revealed that the probiotic administration had favorable effects on HOMA-IR, VLDL, QUICKI, and triglyceride levels in the patients with AD without Hartung-Knapp adjustment. However, the changes in MMSE, RBANS, and other biomarkers of oxidative stress, inflammation, and lipid profiles (hs-CRP, MDA, and total cholesterol) were negligible.

Recently, Tamtaji et al. (27) conducted a systematic review and meta-analysis to evaluate the effects of probiotic supplementation on metabolic status in patients with neurological disorders (including AD, parkinson's disease, multiple sclerosis, and migraine). Tamtaji et al. (27) demonstrated beneficial effects of probiotics on CRP, MDA, insulin, HOMA-IR, triglyceride, VLDL cholesterol, and HDL cholesterol levels in patients with neurological diseases. Compared with the study by Tamtaji et al. (27), we only focused on patients with MCI and AD. The strengths of our meta-analysis included a comprehensive search strategy, supplemented by manual search, bias risk assessment and GRADE certainty of evidence assessment for each outcome. In addition, we used Hartung-Knapp adjustment to provide conservative summary estimates with wide confidence intervals.

The intestinal microbiota is an ecosystem formed by a broad range of symbiotic communities of non-pathogenic microorganisms that exist in the distal end of the human intestine. Gut flora plays an important role in the normal physiological functions of organisms (28). The imbalance of intestinal microbiota, that is, intestinal disorders, is directly related to the origin of various acute and chronic dysfunction processes in the host (29). Therefore, intervention in the gut microbiota is becoming a possible strategy for the treatment of various diseases such as heart failure (HF) (30). Recent preclinical and clinical studies have emphasized the key role of the gut microbiota in cardiovascular health, especially HF prognosis (31). Some probiotic strains (particularly *Lactobacillus, Bifidobacterium*, and *Saccharomyces boulardii*) can be used as adjuvants for conventional treatment of HF (30).

Dementia, especially AD, is a chronic and progressive syndrome that takes more than 20 years to manifest into cognitive impairment (32). Therefore, finding safe drugs to prevent dementia has become a subject of increasing interest. Previous studies showed that probiotic supplementation had beneficial effects on the cognitive function of patients with AD (16), so it might be developed as a dietary supplement for MCI. Dietary habits and regular consumption of functional foods prior to the onset of AD may be beneficial to health and reduce the risk of the disease. The present study showed no significant difference in cognitive function between the trial group and the control group. The results were consistent with those of the study by Krüger et al. (33). Moreover, Ticinesi et al. (34) found that although several observational and interventional studies in animal models of Alzheimer's disease supported the concept of gut–brain regulation of cognitive symptoms, the lack of human data prevented any clinical recommendations on this topic. The effect of probiotics on MCI and moderate AD needs further exploration.

Patients with AD are prone to a variety of complications, such as increased inflammatory markers and oxidative damage, mortality (35), microvascular disease, dyslipidemia, and insulin resistance (36). Current research showed that probiotics could significantly improve HOMA-IR, QUICKI, and triglyceride and VLDL levels in patients with AD, but had no effect on hs-CRP, MDA, and total cholesterol levels. New evidence suggests that brain insulin resistance, as a key mediator of prediabetes and diabetes, might play a role in AD (37). Insulin resistance is important in the development of cognitive dysfunction in elderly patients with essential hypertension (38). On the contrary, the role of lipids in the pathogenesis and progression of AD remains unclear. In a meta-analysis conducted by Kasińska et al. (38), probiotics significantly reduced the HbA1c levels and HOMA-IR in patients with type 2 diabetes, but had no significant effect on fasting glucose, insulin, and lipid profiles. Insulin simulation and lipid-lowering effects of probiotic supplements might be mediated by reducing oxidative stress and pro-inflammatory markers (39), and increasing B-oxidation of long-chain fatty acids in liver and muscle tissue (40). Probiotics can regulate serum lipoproteins by increasing the activities of cholesterol 7α-hydroxylase (CYP7α1), liver X receptor α and CYP7α1 enzyme. This may be the mechanism by which they reduce total cholesterol and triglycerides, increase the production of short-chain fatty acids, and regulate the expression of lipid and sugar genes of glucose-6-polyphosphatase and glucose transporter (40, 41).

This meta-analysis had some limitations that might have affected the interpretation of the results. First, as a protocol has not been pre-registered for this meta-analysis, it may introduce potential bias. Second, the heterogeneity in this study was significant. Due to the small number of studies, subgroup and meta-regression analyses were not possible to be performed to explore the sources of heterogeneity. Third, the number of human participants was limited. Some data were used for two trials, which might lead to bias. Finally, the evaluation of cognitive function lacked consistency and objectivity, and more accurate methods were needed for evaluation.

## Conclusion

In conclusion, the present study demonstrated that the consumption of probiotics had favorable effects on the HOMA-IR in patients with AD. However, the probiotic treatment did not affect cognitive function, other biomarkers of oxidative stress, and other lipid profiles. More randomized controlled trials are warranted to produce robust results and definite conclusions.

## Data Availability Statement

The original contributions presented in the study are included in the article/Supplementary Material, further inquiries can be directed to the corresponding author/s.

## Author Contributions

XL, CL, and JS contributed to conception and design of the study. XL organized the database. CL performed the statistical analysis. JS wrote the first draft of the manuscript. XL, CL, JS, and JL wrote sections of the manuscript. All authors contributed to manuscript revision, read, and approved the submitted version.

## Funding

This study was supported by Key Science and Technology Research Program for Medical Science Research in Hebei Province (20190215).

## Conflict of Interest

The authors declare that the research was conducted in the absence of any commercial or financial relationships that could be construed as a potential conflict of interest.

## Publisher's Note

All claims expressed in this article are solely those of the authors and do not necessarily represent those of their affiliated organizations, or those of the publisher, the editors and the reviewers. Any product that may be evaluated in this article, or claim that may be made by its manufacturer, is not guaranteed or endorsed by the publisher.
