# Supplementary material for: Effect of Probiotic Supplementation on Cognitive Function and Metabolic Status in Mild Cognitive Impairment and Alzheimer's Disease: A Meta-Analysis
Source: Front Nutr. 2021 Dec 8;8:757673. doi: 10.3389/fnut.2021.757673 (PMC8692377; doi:10.3389/fnut.2021.757673)
Supplement: Supplementary file 1 [file Table_1.DOC]

**Table S1 Search strategies for PubMed.**

| **Database** | **Period of Search** | **Search strategies** |
| --- | --- | --- |
| PubMed | February 2021 | ("probiotic s"[All Fields] OR "probiotical"[All Fields] OR "probiotics"[MeSH Terms] OR "probiotics"[All Fields] OR "probiotic"[All Fields]) AND ("alzheimer disease"[MeSH Terms] OR ("alzheimer"[All Fields] AND "disease"[All Fields]) OR "alzheimer disease"[All Fields] OR ("alzheimer s"[All Fields] AND "disease"[All Fields]) OR "alzheimer s disease"[All Fields]) OR "ad"[All Fields]) AND ("probiotics"[All Fields] OR "probiotics"[MeSH Terms] OR "probiotics"[All Fields] OR "probiotic"[All Fields]) AND ("probiotic s"[All Fields] OR "probiotical"[All Fields] OR "probiotics"[MeSH Terms] OR "probiotics"[All Fields] OR "probiotic"[All Fields]) AND ("cognitive dysfunction"[MeSH Terms] OR ("cognitive"[All Fields] AND "dysfunction"[All Fields]) OR "cognitive dysfunction"[All Fields] OR ("mild"[All Fields] AND "cognitive"[All Fields] AND "impairment"[All Fields]) OR "mild cognitive impairment"[All Fields]) AND "MCI"[All Fields] AND ("probiotic s"[All Fields] OR "probiotics"[MeSH Terms] OR "probiotics"[All Fields] OR "probiotic"[All Fields]) |
